# Prevalence of congenital anomalies in newborns: a cross-sectional study in the state of Rio de Janeiro, Brazil, 2019-2021

**DOI:** 10.1590/S2237-96222025v34e20240471.en

**Published:** 2025-05-12

**Authors:** Pauline Lorena Kale, Nina Nogueira Alt, Sandra Costa Fonseca

**Affiliations:** 1 Universidade Federal do Rio de Janeiro, Epidemiologia e Bioestatística, Rio de Janeiro, RJ, Brazil; 2 Médica graduada pela Universidade Federal Fluminense, Niterói, RJ, Brazil; 3 Universidade Federal Fluminense, Departamento de Epidemiologia e Bioestatística, Niterói, RJ, Brazil

**Keywords:** Congenital Abnormalities, Information Systems, Public Health Surveillance, Prenatal Care, Cross-Sectional Studies, Anomalías Congénitas, Sistemas de Información, Vigilancia en Salud Pública, Atención Prenatal, Estudios Transversales

## Abstract

**Objective:**

To describe prevalence of congenital anomalies according to maternal, health care, and newborn characteristics in the state of Rio de Janeiro, from 2019 to 2021.

**Methods:**

This was a cross-sectional study. Live births were described according to sex, birthweight, gestational age, Apgar score, and maternal sociodemographic, reproductive, and health care characteristics. Data were obtained from the Live Birth Information System (Sistema de Informações sobre Nascidos Vivos - Sinasc). Anomalies were classified according to the list of priority congenital anomalies for surveillance within the scope of Sinasc. We calculated prevalence rates and respective 95% confidence intervals (95%CI).

**Results:**

The prevalence rate of congenital anomalies was 68.7/10,000 live births, and was high in children of mothers who were Black (75.9/10,000 live births), <20 years old (74.8 10,000 live births) and ≥35 years old (83.8 10,000 live births), as well as in newborns <1500 g (189.2 10,000 live births) and newborns with gestational age of 22 to 31 weeks (154.8 10,000 live births). The prevalence rate of priority anomalies was 45.8 10,000 live births, twice the prevalence of unclassified anomalies (22.9 10,000 live births). Limb defects predominated, with a prevalence rate of 22.5 10,000 live births (95%CI 21.3; 23.7), followed by heart defects, 6.5 10,000 live births (95%CI 5.9; 7.2). Oral clefts, genital organ anomalies and abdominal wall defects alternated from third to fifth positions.

**Conclusions:**

Newborns with higher biological risk and born to women with greater sociodemographic vulnerability presented higher prevalence of anomalies. The list of priority congenital anomalies should be included in the Sinasc data tabulation programs.

## Resumen

**Objetivo:**

Describir la prevalencia de anomalías congénitas según características maternas, asistenciales y de nacidos vivos en el estado de Río de Janeiro, de 2019 a 2021.

**Métodos:**

Estudio transversal. Los nacidos vivos se describieron según sexo, peso, edad gestacional, Apgar y características maternas sociodemográficas, reproductivas y asistenciales. Los datos fueron obtenidos del Sistema de Información de Nacidos Vivos (Sinasc). Las anomalías fueron clasificadas según la lista de anomalías congénitas prioritarias para la vigilancia en el ámbito del Sinasc. Se calcularon la prevalencia y sus respectivos intervalos de confianza del 95% (IC95%).

**Resultados:**

La prevalencia de anomalías fue de 68,7/10 mil nacidos vivos, alta en hijos de madres negras (75,9/10 mil nacidos vivos), <20 años (74,8 10 mil nacidos vivos) y ≥35 años (83,8 10 mil nacidos vivos), en recién nacidos <1.500 g (189,2 10 mil nacidos vivos) y de 22 a 31 semanas de gestación. (154,8 10 mil nacidos vivos). La prevalencia de anomalías prioritarias fue de 45,8 10 mil nacidos vivos, el doble que la prevalencia de anomalías no clasificadas (22,9 10 mil nacidos vivos). Predominaron los defectos de las extremidades, con una prevalencia de 22,5 10 mil nacidos vivos (IC95% 21,3; 23,7), seguido de las enfermedades cardíacas, 6,5 10 mil nacidos vivos (IC95% 5,9; 7,2). Las fisuras bucales, las anomalías de los órganos genitales y los defectos de la pared abdominal se alternaron de la tercera a la quinta posición.

**Conclusione:**

s: Los recién nacidos con mayor riesgo biológico y los hijos de mujeres con mayor vulnerabilidad sociodemográfica tuvieron mayor prevalencia de anomalías. La lista de anomalías congénitas prioritarias debe insertarse en los programas de tabulación de datos del Sinasc.

## Introduction

Congenital anomalies are the cause of approximately 240,000 deaths per year in newborns and a further 170,000 during childhood (1). Among survivors, serious sequelae and disabilities can occur, impacting families and health services. Middle- and low-income countries account for the majority of cases of congenital anomalies (1). 

Surveillance of congenital anomalies is recommended by the World Health Organization and there are organized systems in several countries, such as the United States Centers for Disease Control and Prevention in the United States, the International Clearinghouse for Birth Defects Surveillance and Research, and the European network of population-based registries for the epidemiological surveillance of congenital anomalies (1).

In Brazil, in 2021, the Ministry of Health proposed a list of priority congenital anomalies for surveillance, considering two criteria: being diagnosable at birth and having preventive intervention at different levels (2). The data source for surveillance of congenital anomalies in Brazil is the Live Birth Information System (*Sistema de Informações sobre Nascidos Vivos* - Sinasc). In addition to maternal sociodemographic, pregnancy, and child delivery information, this system records neonatal characteristics, including structural anomalies visible at the time of delivery (3). The Sinasc is periodically evaluated for data quality and its completeness is excellent (4). Specifically in relation to the congenital anomalies variable, completeness has been heterogeneous across the country (4-5), but in the state of Rio de Janeiro, an improvement in the quality of the variable has been described, with incompleteness of just 2.7% in 2014 (6). Local studies on prevalence and characteristics of congenital anomalies can contribute to knowledge of factors related to this condition (7-8). Consequently, they can qualify prevention measures and support specific planning of care lines, such as the need for tertiary units with a high surgical turnover (9-10). Additionally, measures already in place, such as folic acid supplementation, can be evaluated (11).

As such, the objective of this study was to describe the prevalence of congenital anomalies according to maternal, pregnancy, delivery, reproductive history, healthcare and live birth characteristics in the state of Rio de Janeiro, between 2019 and 2021.

## Methods

### Design

This was a cross-sectional descriptive study of congenital anomalies present at birth, in the state of Rio de Janeiro, from 2019 to 2021. Live births were described both transversally at birth (biological risk factors and anomalies) and also retrospectively (maternal sociodemographic, gestational and healthcare characteristics).

### Setting

In 2022, the population and birth rate of the state of Rio de Janeiro were 16,055,174 and 10.3/1,000 inhabitants, respectively. The Human Development Index (0.762) is high and the state ranks in eighth place in Brazil. 

### Participants

Newborns weighing ≥500 g and/or with a gestational age of ≥22 weeks were eligible, considering the low viability of very immature newborns (12). Newborns outside these criteria were considered ineligible.

### Variables

The description of maternal and newborn variables, as well as their categorization or open field status, are shown in Table 1. The Adequacy of access to prenatal care variable referred to the month in which prenatal care started and the number of prenatal care consultations (13). The congenital anomalie code variable refers to the codes contained in the Tenth Revision of the International Statistical Classification of Diseases and Related Health Problems (ICD-10). 

**Table 1:** Description of maternal and newborn variables with categories or open field

Type of variable	Variables (unit of measurement)	Categories or open field
**Maternal sociodemographic**		
variables	Age (years)	10-19
		20-34
		≥35
		
	Race/skin color	White
		Black
		Mixed race
		
	Years of study	0-3
		4-11
		≥12
		
	Type of Pregnancy	Single
		Multiple
**Pregnancy and Parity**		
variables	Parity	Primiparous-zero
	(number of previous child deliveries)	Multiparous ≥1
		
	Type of delivery	Vaginal
		Cesarean
		
	Adequacy of access to prenatal	No prenatal care
	care (month started=month) and number of consultations=consultations)	Inadequate: month >3, or month ≤3 and consultations <3
		Intermediate: month ≤3 and consultations=from 3 to 5
		Adequate: month ≤3 and consultations=6
		More than adequate: month ≤3 e consultations ≥7
		
	Inadequacy of prenatal care (dichotomization of the Adequacy of access to prenatal care variable)	Yes (no prenatal care, inadequate and intermediate)
		No (adequate and more than adequate)
**Newborn biological**		
variables	Sex	Female
		Male
		
	Birth weight (grams)	<1500
		1500-2499
		2500-3999
		≥4000
		
	Dichotomized birth weight-g	<2500 ≥2500
		
	Gestational age (weeks)	22-31
		32-36
		≥37
		
	Apgar Score at the 5^th^ minute	<7
		≥7
		
	Presence of anomalie	Yes
		No
		
	Congenital anomalie code	Open field

Congenital anomalies were classified according to the list of priority congenital anomalies for surveillance within the scope of Sinasc (2), which selects anomalies from ICD-10 Chapter XVII (Congenital malformations, deformations and chromosomal anomalies) (14), resulting in eight groups:

1) Neural tube defects: anencephaly (Q00.0), craniorachischisis (Q00.1), iniencephaly (Q00.2), encephalocele (Q01) and spina bifida (Q05).
2) Microcephaly (Q02).
3) Congenital heart defects: congenital malformations of the cardiac chambers and connections (Q20), congenital malformations of cardiac septa (Q21), congenital malformations of pulmonary and tricuspid valves (Q22), congenital malformations of aortic and mitral valves (Q23), other congenital malformations of the heart (Q24), congenital malformations of great arteries (Q25), congenital malformations of great veins (Q26), other congenital malformations of the peripheral vascular system (Q27) and other congenital malformations of the circulatory system (Q28).
4) Oral clefts: cleft palate (Q35), cleft lip (Q36) and cleft palate with cleft lip (Q37).
5) Congenital malformations of genital organs: hypospadias (Q54) and indeterminate sex and pseudohermaphroditism (Q56).
6) Limb defects: congenital deformities of feet (Q66), polydactyly (Q69), reduction defects of upper limb (Q71), reduction defects of lower limb (Q72), reduction defects of unspecified limb (Q73) and arthrogryposis multiplex congenita (Q74.3).
7) Abdominal wall defects: exomphalus (Q79.2) and gastroschisis (Q79.3).
8) Down syndrome (Q90).

Congenital anomalies not classified by the list were grouped together and called “Unclassified/other”. 

### Data sources and measurement

The data source is the Sinasc of the Health Department of the state of Rio de Janeiro. The secondary databases were provided for the research without identification of cases. The prevalence rate of the total anomalies is the quotient between the number of newborns with anomalies (field 6 of the Live Birth Certificate), marked “yes”, regardless of having ICD-10 code in field 41 of the Live Birth Certificate) (17), and the total number of live births, multiplied by 10,000. The prevalence of priority congenital anomalies was based on the ICD-10 code, bearing in mind that a same newborn may have anomalies classified in more than one group.

### Study size

From 2019 to 2021, 598,604 live births were recorded, of which 597,535 (99.8%) were eligible for this study.

### Statistical methods

We compared the frequencies of eligible and ineligible newborns according to the completion of field 6 of the Live Birth Certificate. We used Fisher’s exact test.

We calculated the frequency of anomalies per newborn (up to 5), the prevalence rates of total anomalies and those of selected variables and their respective confidence intervals (95%CI). 

## Results

Among the 597,535 eligible newborns, 99.3% of field 6 of the Live Birth Certificate was filled out, 2.6% of them were in the “unknown” category, totaling 3.3% missing information. Among the ineligible (n=1,069), the corresponding values ​​were 99.2%, 3.9% and 4.7%. Differences in the presence of anomalies according to eligibility were significant (<0.005). The prevalence rates of anomalies per 10,000 live births were 68.7 (eligible) and 13.3 (ineligible) (Table 2). 

**Table 2:** Distribution of live births according to presence and frequency of congenital anomalies (fields 6 and 41 of the Live Birth Certificate). Rio de Janeiro, 2019-2021

Fields	2019	2020	2021	Total
	n (%)	n (%)	n (%)	n (%)
**Presence of congenital anomalies**				
Yes	1,415 (0.7)	1,405 (0.7)	1,288 (0.7)	4,108 (0.7)
No	202,196 (97.1)	190,528 (95.6)	181,045 (95.3)	573,769 (96.0)
Unknown	4,379 (2.1)	6,274 (3.2)	4,739 (2.5)	15,392 (2.6)
Left blank	314 (0.2)	1,011 (0.5)	2,941 (1.6)	4,266 (0.7)
Total	208,304 (100.0)	199,218 (100.0)	190,013 (100.0)	597,535 (100.0)
**Frequency of congenital anomalies**				
1 anomalie	1,161 (80.0)	1,157 (80.8)	1,047 (79.0)	3,365 (79.6)
2 anomalies	168 (11.6)	159 (11.1)	159 (12.0)	507 (12.0)
3 anomalies	83 (5.7)	68 (4.7)	81 (6.1)	232 (5.5)
4 anomalies	22 (1.5)	34 (2.4)	22 (1.7)	78 (1.8)
5 anomalies	17 (1.2)	14 (1.0)	17 (1.3)	48 (1.1)
Total	1,451 (100.0)	1,432 (100.0)	1,326 (100.0)	4,230 (100.0)

The results below relate to eligible newborns. 

The prevalence rate of congenital anomalies ranged from 68 to 71/10,000 live births. Frequency of just one anomalie is predominant, ranging from 79% in 2021 to 80.8% in 2020. Frequency of anomalies gradually decreases, reaching 1.1% for the five anomalies category (Table 2).

Newborns of adolescent mothers (<20 years old) and, mainly, older mothers, presented higher prevalence rate of anomalies. Higher prevalence rates were also found among newborns of Black women and women with low levels of education, except in 2019, mothers having multiple pregnancies, those who underwent cesarean section and those who had inadequate prenatal care. The difference between the prevalence rates among primiparous and multiparous mothers was 0.2/10,000 live births. Newborns of the male sex and in situations of biological risk (low birthweight, prematurity and/or asphyxia) also presented higher prevalence rates. The magnitude of the the annual prevalence rate of anomalies varied little, with overlapping of their respective 95% CI (Table 3).

**Table 3:** Absolute total and annual frequency (n) and prevalence of congenital anomalies per 10,000 live births and respective 95% confidence intervals (95%CI), according to maternal, pregnancy, delivery, reproductive history, health care and newborn characteristics. Rio de Janeiro, 2019-2021

Characteristics	2019		2020		2021		Total	
	n	Prevalence (95%CI)	n	Prevalence (95%CI)	n	Prevalence (95%CI)	n	Prevalence (95%CI)
**Age** (years)								
<20	208	72.2 (63.0; 82.6)	209	78.7 (68.8; 90.1)	172	73.5 (63.4; 85.3)	589	74.8 (69.0; 81.1)
20-34	914	63.9 (59.9; 68.1)	884	64.2 (60.1; 68.6)	840	63.3 (59.1; 67.7)	2,638	63.8 (61.4; 66.3)
≥35	293	80.6 (72.0; 90.4)	312	89.1 (79.8; 99.6)	276	81.6 (72.5; 91.7)	881	83.8 (78.4; 89.5)
**Race/skin color**								
White	493	70.7 (64.7; 77.2)	422	66.2 (60.2; 72.8)	370	61.4 (55.5; 68.0)	1,285	66.3 (62.8; 70.0)
Black	189	68.9 (59.9; 79.5)	226	80.7 (70.9; 91.9)	221	81.0 (71.1; 92.4)	636	76.9 (71.2; 83.1)
Mixed race	674	63.0 (58.4; 67.9)	699	68.5 (63.6; 73.8)	648	66.9 (62.0; 72.2)	2,021	66.1 (63.3; 69.0)
**Years of study**								
<4	16	57.1 (35.2; 92.5)	19	76.6 (49.1; 119.3)	21	99.2 (65.0; 151.2)	56	75.7 (58.3; 98.1)
4-11	1,063	67.3 (63.4; 71.5)	1,069	70.6 (66.5; 75.0)	1,002	70.3 (66.1; 74.8)	3,134	69.4 (67.0; 71.9)
≥12	294	67.2 (59.9; 75.3)	291	70.2 (62.6; 78.7)	241	58.5 (51.6; 66.3)	826	65.3 (61.0; 69.9)
**Pregnancy**								
Single	1,371	67.4 (63.9; 71.0)	1,359	69.8 (66.2; 73.6)	1,262	68.0 (64.4; 71.9)	3,992	68.4 (66.3; 70.6)
Multiple	41	86.2 (63.6; 116.7)	46	102.2 (76.7; 136.0)	24	54.9 (36.9; 81.5)	111	81.4 (67.7; 98.0)
**Parity**								
Primiparous	645	71.9 (66.5; 77.6)	547	65.9 (60.6; 71.6)	540	67.1 (61.7; 73.0)	1,732	68.4 (65.3; 71.7)
Multiparous	761	65.3 (60.8; 70.0)	802	71.3 (66.6; 76.4)	734	68.0 (63.3; 73.1)	2,297	68.2 (65.4; 71.0)
**Type of delivery**								
Vaginal	466	53.1 (48.5; 58.1)	463	56.1 (51.2; 61.4)	423	53.4 (48.6; 58.7)	1,352	53.4 (50.6; 56.3)
Cesarean	945	78.5 (73.7; 83.7)	942	80.8 (75.9; 86.1)	865	78.1 (73.1; 83.5)	2,752	81.7 (78.7; 84.8)
**Prenatal**								
Adequate	918	63.4 (59.5; 67.7)	859	62.9 (58.8; 67.2)	810	61.2 (57.2; 65.6)	2,587	62.5 (60.2; 65.0)
Inadequate	377	76.7 (69.4; 84.8)	426	88.1 (80.2; 96.9)	380	85.7 (77.5; 94.7)	1,183	83.4 (78.8; 88.3)
**Sex**								
Female	588	57.6 (53.2; 62.5)	587	60.4 (55.8; 65.5)	538	57.8 (53.1; 62.9)	1,713	58.6 (55.9; 61.5)
Male	809	76.2 (71.1; 81.6)	796	78.0 (72.8; 83.6)	728	75.1 (68.9; 80.8)	2,333	76.5 (73.4; 79.6)
**Weight** (grams)								
<1500	72	238.9 (190.1; 299.8)	87	291.9 (237.3; 358.7)	85	302.5 (245.3; 372.5)	244	277.1 (244.9; 313.5)
1500-2499	296	185.0 (165.3; 207.1)	272	177.0 (157.3; 199.0)	283	187.7 (167.2; 210.6)	851	183.2 (171.4; 195.8)
2500-3999	984	55.1 (51.8; 58.6)	985	58.0 (54.5; 61.7)	868	53.4 (50.0; 57.0)	2,837	55.5 (53.5; 57.6)
≥4000	63	59.1 (46.2; 75.6)	61	55.8 (43.4; 71.6)	52	54.6 (41.7; 71.5)	176	56.6 (48.8; 65.5)
**Pregnancy** (weeks)								
22-31	61	189.1 (147.5; 242.2)	58	181.2 (140.4; 233.5)	59	198.0 (153.8; 254.5)	178	189.2 (163.6; 218.8)
32-36	271	140.9 (125.1; 158.5)	316	168.2 (150.8; 187.6)	285	155.7 (138.7; 174.6)	872	154.8 (144.9;165.3)
≥37	1,061	57.8 (54.4; 61.4)	1,011	58.0 (54.5; 61.7)	922	55.7 (52.2; 59.4)	2,994	57.2 (55.2; 59.3)
**Apgar ^a^t 5th minute**								
<7	151	717.3 (614.7; 835.5)	140	672.8 (572.9; 788.5)	144	712.9 (608.6; 833.4)	435	700.9 (640.0; 767.1)
≥7	1,246	61.1 (57.8; 64.6)	1,249	64.0 (60.6; 67.7)	1,136	61.0 (57.6; 64.7)	3,631	62.1 (60.1; 64.1)

In the three-year period, among the 4,108 newborns with anomalies that were described, 2,739 (66.7%) were classified as per the priority anomalies list, corresponding to a prevalence rate of 45.8/10,000 live births, this being twice the prevalence of unclassified anomalies (22.9/10,000 live births). The prevalence pattern of congenital priority anomalies is the same each year. Higher values ​​were found for limb defects, 3.8 to 4.6 times the annual value of the second most prevalent defect, i.e. congenital heart defects, in all years analyzed, except in 2021, when oral clefts surpassed heart defects. In the other years, the third, fourth, and fifth most prevalent defects alternate between oral clefts, genital organ anomalies, and abdominal wall defects (Table 4).

**Table 4:** Absolute frequency (n) of congenital anomalies and respective prevalence rates per 10,000 live births, with 95% confidence intervals (95%CI), according to the list of priority congenital anomalies for surveillance within the scope of the Live Birth Information System. Rio de Janeiro, 2019-2021

Congenital anomalies	2019		2020		2021		Total	
	n	Prevalence (95%CI)	n	Prevalence (95%CI)	n	Prevalence (95%CI)	n	Prevalence (95%CI)
**Priority anomalies**								
1) Neural tube defects	71	3.4 (2.7; 4.3)	71	3.6 (2.8; 4.5)	72	3.8 (3.0; 4.8)	214	3.6 (3.1; 4.1)
2) Microcephaly	12	0.6 (0.3; 1.0)	19	1.0 (0.6; 1.5)	18	0.9 (0.6; 1.5)	49	0.8 (0.6; 1.1)
3) Congenital heart defects	116	5.6 (4.6; 6.7)	120	6.0 (5.0; 7.2)	92	4.8 (3.9; 5.9)	328	6.5 (5.9; 7.2)
4) Oral clefts	86	4.1 (3.3; 5.1)	89	4.5 (3.6; 5.5)	95	5.0 (4.1; 6.1)	270	4.5 (4.0; 5.1)
5) Genital organ malformations	94	4.5 (3.7; 5.5)	86	4.3 (3.5; 5.3)	72	3.8 (3.0; 4.8)	252	4.2 (3.7; 4.8)
6) Limb defects	446	21.4 (19.5; 23.5)	463	23.2 (21.2; 25.5)	434	22.8 (20.8; 25.1)	1,343	22.5 (21.3; 23.7)
7) Abdominal wall defects	86	4.1 (3.3; 5.1)	88	4.4 (3.6; 5.4)	70	3.7 (2.9; 4.7)	244	4.1 (3.6; 4.6)
8) Down syndrome	56	2.7 (2.1; 3.5)	62	3.1 (2.4; 4.0)	66	3.5 (2.7; 4.4)	184	3.1 (2.7; 3.6)
**At least one priority anomalie**	924	44.4 (41.6; 47.3)	949	47.6 (44.7; 50.8)	866	45.6 (42.7; 48.7)	2,739	45.8 (44.2; 47.6)
**Unclassified anomalie**	491	23.6 (21.6; 25.8)	456	22.9 (20.9; 25.1)	422	22.2 (20.2; 24.4)	1,369	22.9 (21.7; 24.2)

Considering that there were small variations in the annual prevalence rates of anomalies, the following results are presented only for the three-year period (Table 5).

**Table 5:** Absolute (n) and percentage (%) distribution of live births with congenital anomalies and without anomalies, according to the list of priority congenital anomalies for surveillance within the scope of the Live Birth Information System, by maternal, pregnancy, delivery, reproductive history, health care and newborn characteristics. Rio de Janeiro, 2019-2021

Characteristics	Neural tube defect n (%)	Microcephaly n (%)	Heart defects n (%)	Oral clefts n (%)	Genital organs n (%)	Limbs n (%)	Abdominal wall n (%)	Down syndrome n (%)	No anomalie n (%)
**Age** (years)									
<20	24 (11.2)	8 (16.3)	26 (7.9)	24 (8.9)	39 (15.5)	195 (14.5)	88 (36.1)	10 (5.4)	76,129 (13.3)
20-34	144 (67.3)	32 (65.3)	204 (62.2)	179 (66.3)	159 (63.1)	925 (68.9)	133 (54.5)	63 (34.2)	398,127 (69.4)
≥35	46 (21.5)	9 (18.4)	98 (29.9)	67 (24.8)	54 (21.4)	223 (16.6)	23 (9.4)	111 (60.3)	99,491 (17.3)
**Race/skin color**									
White	64 (33.2)	21 (45.7)	143 (46.3)	92 (35.0)	79 (32.0)	352 (26.8)	60 (27.8)	85 (48.0)	184,901 (32.9)
Black	30 (15.5)	7 (15.2)	40 (12.9)	34 (12.9)	45 (18.2)	251 (19.1)	31 (14.4)	16 (9.0)	80,396 (14.3)
Mixed race	99 (51.3)	18 (39.1)	126 (40.8)	137 (52.1)	123 (49.8)	711 (54.1)	125 (57.9)	76 (42.9)	296,344 (52.8)
**Years of study**									
<4	5 (2.4)	0	1 (0.3)	3 (1.1)	3 (1.2)	18 (1.4)	1 (0.4)	2 (1.1)	7,216 (1.3)
4-11	158 (76.0)	44 (89.8)	195 (60.4)	201 (75.6)	203 (82.5)	1,074 (81.8)	216 (88.9)	111 (62.0)	437,095 (77.6)
≥12	45 (21.6)	5 (10.2)	127 (39.3)	62 (23.3)	40 (16.3)	221 (16.8)	26 (10.7)	66 (36.9)	118,747 (21.1)
**Pregnancy**									
Single	205 (95.8)	49 (100.0)	316 (96.3)	268 (99.3)	245 (97.2)	1,314 (97.9)	235 (96.3)	181 (98.4)	560,452 (97.7)
Multiple	9 (4.2)	0	12 (3.7)	2 (0.7)	7 (2.8)	28 (2.1)	9 (3.7)	3 (1.6)	13,069 (2.3)
**Parity**									
Primiparous	82 (39.4)	19 (40.4)	155 (48.1)	98 (36.6)	132 (52.6)	569 (42.7)	137 (59.8)	67 (36.6)	243,823 (42.9)
Multiparous	126 (60.6)	28 (59.6)	167 (51.9)	170 (63.4)	119 (47.4)	765 (57.3)	92 (40.2)	116 (63.4)	324,229 (57.1)
**Type of delivery**									
Vaginal	48 (22.4)	11 (22.4)	54 (16.5)	86 (33.5)	90 (33.5)	547 (33.5)	55 (33.5)	47 (33.5)	241,946 (33.5)
Cesarean	166 (77.6)	38 (77.6)	274 (83.5)	184 (63.4)	162 (63.4)	793 (63.4)	189 (63.4)	137 (63.4)	331,572 (63.4)
**Prenatal**									
Adequate	117 (61.3)	28 (60.9)	250 (80.6)	182 (73.1)	160 (69.9)	825 (67.0)	127 (56.4)	143 (81.3)	399,007 (74.4)
Inadequate	74 (38.7)	18 (39.1)	60 (19.4)	67 (26.9)	69 (30.1)	406 (33.0)	98 (43.6)	33 (18.8)	137,258 (25.6)
**Sex**									
Female	111 (52.9)	29 (60.4)	166 (50.9)	11 (6.6)	4 (2.1)	531 (39.9)	101 (41.9)	92 (50.0)	281,052 (49.0)
Male	99 (47.1)	19 (39.6)	160 (49.1)	156 (93.4)	186 (97.9)	799 (60.1)	140 (58.1)	92 (50.0)	292,717 (51.0)
**Weight** (grams)									
<1500	33 (15.4)	3 (6.1)	28 (8.5)	14 (5.2)	27 (10.7)	43 (3.2)	23 (9.4)	3 (1.6)	8,307 (1.4)
1500-2499	56 (26.2)	19 (38.8)	78 (23.8)	51 (18.9)	70 (27.8)	147 (10.9)	129 (52.9)	37 (20.1)	44,174 (7.7)
≥2500	125 (58.4)	27 (55.1)	222 (67.7)	205 (75.9)	155 (61.5)	1,153 (85.9)	92 (37.7)	144 (78.3)	521,288 (90.9)
**Pregnancy** (weeks)									
22-31	22 (10.5)	1 (2.1)	13 (4.0)	9 (3.4)	19 (7.7)	36 (2.7)	20 (8.4)	3 (1.6)	8,876 (1.6)
32-36	45 (21.4)	14 (29.2)	87 (27.1)	44 (16.5)	61 (24.6)	178 (13.4)	105 (44.1)	41 (22.4)	53,595 (9.5)
≥37	143 (68.1)	33 (68.8)	221 (68.8)	214 (80.1)	168 (67.7)	1,115 (83.9)	113 (47.5)	139 (76.0)	504,495 (89.0)
**A^pg^ar 5th minute**									
<7	59 (28.0)	15 (31.3)	43 (13.1)	23 (8.6)	33 (13.3)	67 (5.0)	32 (13.2)	5 (2.7)	5,569 (1.0)
≥7	152 (72.0)	33 (68.8)	284 (86.9)	244 (91.4)	215 (86.7)	1,263 (95.0)	210 (86.8)	178 (97.3)	562,317 (99.0)

Among newborns with anomalies that are a priority for reporting and those without anomalies, their mothers were predominantly aged between 20 and 34, followed by those aged 35 years and over. The exceptions were newborns with abdominal wall defects, for whom adolescent mothers (36.1%) were more frequent; and those with Down syndrome, for whom mothers aged ≥35 years predominated (60.3%). There was a higher frequency of mothers of mixed race in the overall results, except in cases of newborns with microcephaly or heart defects, for whom the frequency of mothers of White race/skin color was higher than that of mixed race mothers (Table 4). 

The mothers of the newborns predominantly had between 4 and 11 years of study, regardless of the presence of anomalie, followed by those who had ≥12 years of study. It can be seen that mothers of newborns with heart defects and Down syndrome have higher percentages of higher education (39.3% and 36.9%, respectively), while mothers of newborns with microcephaly and abdominal wall defects have much lower percentages in this category (10.2% and 10.7%, respectively), and mothers of newborns with neural tube defects have the highest percentage of low education (2.4%) (Table 5).

Single pregnancies were the most frequent, regardless of the presence or type of anomalie classified by the list of priority anomalies. No mother of a newborn child with microcephaly had multiple pregnancies. Primiparous pregnancies predominated only among newborns with genital organ anomalies and abdominal wall defects (Table 5).

Cesarean sections were more frequent, regardless of the presence of anomalies, with higher percentages among women whose newborns had heart defects, neural tube defects and microcephaly. The highest frequencies of inadequate prenatal care occurred among mothers of newborns with abdominal wall defects, neural tube defects and microcephaly (Table 5).

The most anomalies were more frequent in boys, except for neural tube defects, microcephaly and heart defects, which were more common in girls, while Down syndrome showed no difference between boys and girls. Genital anomalies stood out, almost all among boys. Frequency of low birth weight was high in all priority anomalie groups, while among those with neural tube defects, genital organ anomalies and abdominal wall defects the frequency of birth weight <1500g was higher. Gestational age <37 weeks was also more frequent among those with anomalie. The highest frequency of prematurity was recorded in newborns with abdominal wall defects, genital anomalies, neural tube defects and heart defects. Asphyxia accounted for a high proportion among newborns with microcephaly and neural tube defects (25.1%). Among newborns with no anomalies, asphyxia accounted for 1% (Table 5).

Of the 4,108 newborns with anomalies, 34.1% (1,369) were not classified according to the list of priority anomalies. Most unclassified anomalies were single anomalies (n=1,119; 81.7%), the most frequent being: multiple congenital malformations, not elsewhere classified (Q89.7; n=83), unspecified congenital hydrocephalus, unspecified (Q03.9; n=58), congenital diaphragmatic hernia (Q79.0; n=40), congenital malformation, unspecified (Q89.9; n=37), and congenital malformation of the ear, unspecified (Q17.9; n=36). Six newborns presented an abnormality contained in ICD-10 Chapter II (Neoplasms), namely D18.0 – haemangioma, any site).

## Discussion

In Rio de Janeiro, from 2019 to 2021, the prevalence of anomalies was higher in newborns with higher biological risk (low birthweight, prematurity and asphyxia), children born to women with greater sociodemographic vulnerability (adolescents/older women, women of Black race/skin color and women with low levels of education) and who had inadequate prenatal care. Considering the level of development of the state of Rio de Janeiro, social inequalities and inequalities in access to health services were identified in the prevalence of anomalies.

The multiple relationships between the variables and potential confounders, not taken into consideration, in addition to the cross-sectional nature of this study, limit causal interpretation of the results. Regarding the validity of the diagnosis of congenital anomalie, it is known that the more complex the anomalie, the greater the risk of underreporting and incomplete data (15). The list of priority anomalies includes most congenital anomalies, however, it has not yet been incorporated into the data tabulation applications provided by the Brazilian National Health System Department of Information Technology, this being a limiting factor for its use.

In Brazil as a whole and specifically in the country’s Southeast region (where the state of Rio de Janeiro is located), from 2001 to 2018, the trend of anomalie prevalence rate showed an annual growth of 5.2% and 5.7%, respectively. This increase is attributed to the version of the Live Birth Certificate in force since 2011, as it allows greater collection of information on anomalie, as well as being attributed to the Zika virus epidemic from 2015 to 2016 (16). The anomalie prevalence rate per 10,000 live births was 87.2 for Brazil as a whole, from 2018 to 2020 (3), and 93.0 in the state of Rio Grande do Sul, from 2012 to 2015 (7), these being values ​​higher than those of the state of Rio de Janeiro found in our study. In turn, in Tangará da Serra, in the state of Mato Grosso, prevalence was lower, i.e. approximately 49/10,000 live births (17). Regarding the prevalence rate of priority anomalies, the rates ​​for Rio Grande do Sul from 2010 to 2019 were also higher than those for Rio de Janeiro from 2019 to 2021, i.e. 60.7/10,000 live births (18).

Among newborns with anomalies analyzed in this study, 79.6% had only one anomalie, a rate close to that for Brazil as a whole from 2018 to 2020 (81.3%) (3). 

Regarding the profile of newborns with anomalies, most of the results found converged with those of other national studies based on Sinasc data. The highest prevalence rates in Brazil as a whole correspond to live borns to women of Black race/skin color, aged >30 years, with <7 prenatal consultations, male newborns weighing <1500g, premature, with asphyxia (3). These are results similar to those we found for the state of Rio de Janeiro. In the state of Santa Catarina, from 2010 to 2018, and in the city of São Paulo-SP, from 2010 to 2014, occurrence of anomalies was similarly associated with low birthweight, prematurity, low Apgar score (19-20) and no prenatal consultations (7). With regard to maternal schooling, we found higher prevalence among those who had <4 years of study, this being in agreement with anomalie prevalence odds ratio estimated in Rio Grande do Sul (<4 years of study compared with to those with ≥12 years of study) (7). In contrast, in Brazil as a whole and in Tangará da Serra, higher anomalie prevalence rates occurred among mothers with ≥12 years of schooling (3,17). Few studies have been found that used the list of priority anomalies (18).

Limb anomalies are characterized by absence or severe hypoplasia of a complete limb or part of it. Coexistence of other anomalies is also common (18). In the state of Rio de Janeiro, limb defects stood out as the main group on the list, with a prevalence rate of 22.5/10,000 live births, this rate being 3.5 times that of heart defects, which came in second place. It should be noted that limb defects are very easy to diagnose at birth, which is not always the case with heart defects (2). Among newborns with limb defects, those with lower biological risk (weight ≥2500g, full-term and without asphyxia) were predominant. 

Heart defects are the most common anomalies, although they may be underestimated when identified at birth (18). Preexisting maternal diseases, such as diabetes and collagen diseases, as well as those acquired during pregnancy, such as viral and parasitic infections, medications, behavioral factors (use of legal and illegal drugs) and advanced maternal age are among maternal risk factors for heart defects (18). Among these factors mentioned here, only information on maternal age is available on the Sinasc. In the state of Rio de Janeiro, among newborns with heart defects reported at birth, 30% of mothers were ≥35 years old, and in the city of São Paulo-SP, the highest prevalence rate of heart defects was also recorded in this age group (21). The prevalence rate of heart defects per 10,000 live births in Brazil as a whole ranged from 5.1 to 10.8, and in the state of Rio de Janeiro, it ranged from 2.9 to 5.7, from 2010 to 2019 (18). In the state of São Paulo, it was as high as 12.4/10,000 live births, with an upward trend (21). Our results showed a heart defect prevalence rate of 6.5/10,000 live births, and the profile of newborns with heart defects was similar to that of Brazil as a whole (18).

The order of priority anomalie frequency was as follows: oral clefts, genital organ anomalies and abdominal wall defects, with similar prevalence rates, i.e. 4.5, 4.2 and 4.1/10,000 live births, respectively.

Oral clefts are common among anomalies and are generally diagnosed by physical examination at birth, although they can be detected by ultrasound during prenatal care (18). In the state of Rio de Janeiro, a gradual increase in the annual prevalence of oral clefts was found, especially in 2020 and 2021 (COVID-19 pandemic), when compared to 2019, although with overlapping 95% CIs. A similar pattern was found with prevalence of neural tube anomalies, which came in fifth place. Problems with prenatal care during the pandemic may have affected preventive measures having been performed (22). Investigation of the consequences of SARS-COV-19 infection for fetuses during pregnancy is still ongoing. Particularly in relation to oral clefts, a retrospective and controlled hospital-based study found association between nonsyndromic cleft palate in newborns and COVID-19 infection during pregnancy, but that analysis was not adjusted for confounding factors (20).

Genital organ anomalies relate to disorders of sexual differentiation (18). In Brazil as a whole, the prevalence rate was 4.7/10,000 live births (from 2010 to 2019) and the maternal profile was similar to that of the state of Rio de Janeiro from 2019 to 2021: young, mixed race/skin color mothers with high school education. In the Federal District, from 2010 to 2021, this group reached came in second place (10.3/10,000 live births), after limb defects (21.8/10,000 live births) (23). Underreporting of sexual organ anomalies has been reported in some Brazilian publications when compared to European rates ​​(18,24).

Abdominal wall defects are characterized by abdominal organ herniation (18). They can be diagnosed during prenatal care and are visible at birth (25). A known risk factor for these anomalies is young maternal age (9,26). In our study, we found that newborns of adolescent mothers had higher prevalence of abdominal wall defects when compared to other priority anomalie groups and groups without anomalies.

The priority anomalie group with the lowest prevalence rate in this study was microcephaly (0.8/10,000 live births). Only severe microcephaly (head circumference <3 standard deviations) is reported on the Sinasc (27). In Brazil as a whole, and particularly in the state of Rio de Janeiro, the annual prevalence rate of microcephaly per 100,000 live births, from 2010 to 2019, was less than 1, except in 2015 and 2016 (Zika virus epidemic), when the rates ​​were around 6 and 8 for Brazil and 2.1 and 10.6 in for the state of Rio de Janeiro, respectively (18). In Brazil as a whole, the characteristics of mothers and newborns with microcephaly were consistent with those found in this study from 2019 to 2021, with the exception of the higher prevalence of mothers of mixed race/skin color nationally compared to mothers of White race/skin color in the state of Rio de Janeiro, as well as higher incidence of asphyxia, which was 12.2% for Brazil as a whole and approximately 31% for the state of Rio de Janeiro (18).

Inclusion of microcephaly on the list of priority anomalies was due to the fact that it is a sentinel anomalie for congenital infections, such as Zika virus and cytomegalovirus (2). In July 2024, the National International Health Regulations Focal Point in Brazil informed the Pan American Health Organization (PAHO) about the suspected vertical transmission of the Oropouche virus (OROV) (28). In Brazil, retrospective investigation of serum and cerebrospinal fluid samples was carried out with negative results for dengue, chikungunya, Zika, and West Nile virus. OROV IgM antibodies were detected in the serum and cerebrospinal fluid of four newborns with microcephaly (28). As such, PAHO has requested that Member States with proven transmission of OROV or other arboviruses intensify surveillance in pregnant women and report the occurrence of spontaneous abortion/fetal death associated with OROV infection, as well as their increase and congenital malformations in newborns that cannot be explained by a known cause (28).

Down syndrome is the most common genetic syndrome and presents signs and symptoms that are generally recognized at birth, with some being able to be diagnosed during prenatal care (2,18). Advanced maternal age increases the risk of chromosomal anomalies, including Down syndrome (1), and its occurring together with congenital heart defects is common (18). In this study, the highest frequency of mothers ≥35 years old was found only for newborns with Down syndrome (60.3%), when compared to the other groups and those without anomalies.

Measures to prevent congenital anomalies include preconception care for women with chronic health conditions that may affect embryogenesis or fetal development, prenatal care, and multidisciplinary health care for children with anomalies (1,29).

The importance of monitoring congenital anomalies using Sinasc data and analyzing them to produce qualified information is essential for targeting health actions, especially considering the constant improvement of Sinasc. As a strong point of the study, we highlight the excellent completeness of fields 6 and 41 of the Live Born Certificate, this being in keeping with what was found for Brazil as a whole, despite regional differences (30). In view of the alert of the possible relationship between OROV infection and anomalies, monitoring of anomalies by health information systems should be intensified. The list of priority anomalies expands knowledge of anomalies and assists in health actions, and should be implemented in data tabulation programs for routine use by health workers, researchers and managers.

We conclude that this study identified the prevalence and sociodemographic characteristics of congenital anomalies in the state of Rio de Janeiro, which had not been described in recent years. The pattern found was similar to the national one regarding the order of frequency of congenital anomalies and the inequalities in their occurrence. This study will serve as a baseline for monitoring prevalence of priority anomalies in the state of Rio de Janeiro.

## Data Availability

The data used in this study are publicly accessible and are provided by the Rio de Janeiro State Health Department.
