# Supplementary material for: Prevalence of congenital anomalies in newborns: a cross-sectional study in the state of Rio de Janeiro, Brazil, 2019-2021
Source: Epidemiol Serv Saude. 2025 May 12;34:e20240471. doi: 10.1590/S2237-96222025v34e20240471.en (PMC12077545; doi:10.1590/S2237-96222025v34e20240471.en)
Supplement: Translation

# Translation: Prevalência de anomalias congênitas em nascidos vivos: um estudo transversal no estado do Rio de Janeiro, 2019-2021

**DOI:** 10.1590/S2237-96222025v34e20240471.pt

**Authors:** Pauline Lorena Kale, Nina Nogueira Alt, Sandra Costa Fonseca

**Affiliations:** 1 Universidade Federal do Rio de Janeiro, Epidemiologia e Bioestatística, Rio de Janeiro, RJ, Brasil; 2 Médica graduada pela Universidade Federal Fluminense, Niterói, RJ, Brasil; 3 Universidade Federal Fluminense, Departamento de Epidemiologia e Bioestatística, Niterói, RJ, Brasil

**Keywords:** Anormalidades congênitas, Sistema de Informação, Vigilância em Saúde Pública, Cuidado Pré-natal, Estudos de Prevalência

## Abstract

**Objetivo:**

Descrever a prevalência de anomalias congênitas segundo características maternas, assistenciais, e dos recém-nascidos no estado do Rio de Janeiro, de 2019 a 2021.

**Métodos:**

Estudo transversal. Nascidos vivos foram descritos segundo sexo, peso, idade gestacional, Apgar e características maternas sociodemográficas, reprodutivas e assistenciais. Dados obtidos no Sistema de Informações sobre Nascidos Vivos (Sinasc). As anomalias foram classificadas segundo a lista de anomalias congênitas prioritárias para vigilância no âmbito do Sinasc. Foram calculados as prevalências e os respectivos intervalos de confiança de 95% (IC95%).

**Resultados:**

A prevalência de anomalias foi 68,7/10 mil nascidos vivos, elevada em filhos de mães pretas (75,9/10 mil nascidos vivos), <20 anos (74,8 10 mil nascidos vivos) e ≥35 anos (83,8 10 mil nascidos vivos), em recém-nascidos <1.500 g (189,2 10 mil nascidos vivos) e de 22 a 31 semanas de gestação (154,8 10 mil nascidos vivos). A prevalência de anomalias prioritárias foi 45,8 10 mil nascidos vivos, duas vezes superior à prevalência das anomalias não classificadas (22,9 10 mil nascidos vivos). Predominaram defeitos de membros, cuja prevalência foi 22,5 10 mil nascidos vivos (IC95% 21,3; 23,7), seguidos pelas cardiopatias, 6,5 10 mil nascidos vivos (IC95% 5,9; 7,2). Fendas orais, anomalias de órgãos genitais e defeitos de parede abdominal alternaram da terceira à quinta posições.

**Conclusões:**

Recém-nascidos com maior risco biológico e filhos de mulheres com maior vulnerabilidade sociodemográfica apresentaram maiores prevalências de anomalias. A lista de anomalias congênitas prioritárias deve ser inserida nos programas de tabulações de dados do Sinasc.

## Introdução

As anomalias congênitas acarretam cerca de 240 mil mortes anuais em recém-nascidos e outras 170 mil durante a infância (1). Entre os sobreviventes, podem ocorrer sequelas e deficiências graves, impactando famílias e serviços de saúde. Países de média e baixa renda concentram a maioria dos casos de malformações congênitas (1). 

A vigilância de anomalias congênitas é recomendada pela Organização Mundial da Saúde e existem sistemas organizados em diversos países, como o Centro de Controle e Prevenção de Doenças dos Estados Unidos, o International Clearinghouse for Birth Defects Surveillance and Research, e o *European network of population-based registries for the epidemiological surveillance of congenital anomalies* (1).

No Brasil, o Ministério da Saúde propôs, em 2021, uma lista de anomalias congênitas prioritárias para vigilância, considerando-se dois critérios: ser diagnosticável ao nascimento e possuir intervenção preventiva em diferentes níveis (2).

A fonte de dados para a vigilância das anomalias congênitas no Brasil é o Sistema de Informações sobre Nascidos Vivos (Sinasc). Este sistema, além de informações sociodemográficas maternas, da gestação e do parto, registra características neonatais, incluindo anomalias estruturais visíveis no momento do parto (3). 

O Sinasc é avaliado periodicamente quanto à qualidade dos dados e sua completude é excelente (4). Especificamente para a variável anomalias congênitas, a completude tem sido heterogênea no país (4-5), mas, no estado do Rio de Janeiro, foi descrita melhora da qualidade da variável, com incompletude de apenas 2,7% em 2014 (6). Estudos locais sobre a prevalência e características das anomalias congênitas podem contribuir para o conhecimento de fatores relacionados a este agravo (7-8). Consequentemente, podem qualificar as medidas de prevenção e subsidiar a programação específica das linhas de cuidado, como, por exemplo, a necessidade de unidades terciárias de grande volume cirúrgico (9-10). Adicionalmente, podem ser avaliadas medidas já instituídas, como a suplementação de ácido fólico (11).

Nesse sentido, o presente trabalho teve como objetivo descrever a prevalência de anomalias congênitas segundo características maternas, da gestação, do parto, da história reprodutiva, assistenciais e dos nascidos vivos no estado do Rio de Janeiro, entre 2019 e 2021.

## Métodos

### Delineamento

Estudo transversal descritivo das anomalias congênitas presentes ao nascimento, no estado do Rio de Janeiro, de 2019 a 2021. Nascidos vivos foram descritos transversalmente ao nascimento (fatores de risco biológico e anomalias) e retrospectivamente (características maternas sociodemográficas, gestacionais e assistenciais).

### Contexto

Em 2022, a população e a taxa de natalidade do estado do Rio de Janeiro foram 16.055.174 e 10,3/1 mil habitantes. O Índice de Desenvolvimento Social (0,762) é alto e o estado ocupa a oitava posição do país. 

### Participantes

Foram elegíveis os recém-nascidos com peso ≥500g e/ou idade gestacional ≥22 semanas, considerando-se a baixa viabilidade dos neonatos muito imaturos (12). Os demais recém-nascidos foram considerados não elegíveis.

### Variáveis

A descrição das variáveis maternas e do recém-nascido, assim como sua categorização ou campo aberto, encontram-se na Tabela 1. A variável adequação de acesso ao pré-natal considerou o mês de início e o número de consultas (13). A variável código da anomalia congênita refere-se ao código da Décima Revisão da Classificação Estatística Internacional de Doenças e Problemas Relacionados à Saúde (CID-10). 

**Tabela 1:** Descrição das variáveis maternas e dos recém-nascidos com categorias ou campo aberto

Tipo de variáveis	Variáveis (unidade de medida)	Categorias ou campo aberto
**Sociodemográficas maternas**		
	Idade (anos)	10-19
		20-34
		≥35
		
	Raça/cor da pele	Branca
		Preta
		Parda
		
	Anos de estudo	0-3
		4-11
		≥12
		
	Tipo de Gravidez	Única
		Múltipla
**Gravidez e Paridade**		
	Paridade	Primípara-zero
	(número de partos anteriores)	Multípara ≥1
		
	Tipo de parto	Vaginal
		Cesariana
		
	Adequação de acesso ao pré-	Não fez
	natal (mês de início=mês) e número de consultas=consultas)	Inadequado: mês >3, ou mês ≤3 e consultas <3
		Intermediário: mês ≤3 e consultas de 3 a 5
		Adequado: mês ≤3 e consultas=6
		Mais do que adequado: mês ≤3 e consultas ≥7
		
	Inadequação do pré-natal (dicotomização da variável Adequação de acesso ao pré-natal)	Sim (não fez, inadequado e intermediário)
		Não (adequado e mais do que adequado)
**Biológicas dos recém**-nascidos		
	Sexo	Feminino
		Masculino
		
	Peso ao nascer (gramas)	<1.500
		1.500-2.499
		2.500-3.999
		≥4.000
		
	Peso ao nascer dicotomizada-g	<2.500 ≥2.500
		
	Idade gestacional (semanas)	22-31
		32-36
		≥37
		
	Apgar no 5º minuto	<7
		≥7
		
	Presença de anomalia	Sim
		Não
		
	Código da anomalia congênita	Campo aberto

As anomalias congênitas foram classificadas segundo a lista de anomalias congênitas prioritárias para vigilância no âmbito do Sinasc (2), que seleciona anomalias do capítulo XVII (Malformações congênitas, deformidades e anomalias cromossômicas) da CID-10 (14), resultando em oito grupos:

1) Defeitos do tubo neural: anencefalia (Q00.0), craniorraquisquise (Q00.1), iniencefalia (Q00.2), encefalocele (Q01) e espinha bífida (Q05).
2) Microcefalia (Q02).
3) Cardiopatias congênitas: malformações congênitas das câmaras e das comunicações cardíacas (Q20), malformações congênitas dos septos cardíacos (Q21), malformações congênitas das valvas pulmonar e tricúspide (Q22), malformações congênitas das valvas aórtica e mitral (Q23), outras malformações congênitas do coração (Q24), malformações congênitas das grandes artérias (Q25), malformações congênitas das grandes veias (Q26), outras malformações congênitas do sistema vascular periférico (Q27) e outras malformações congênitas do aparelho circulatório (Q28).
4) Fendas orais: fenda palatina (Q35), fenda labial (Q36) e fenda labial com fenda palatina (Q37).
5) Anomalias de órgãos genitais: hipospádia (Q54) e sexo indeterminado e pseudo-hermafroditismo (Q56).
6) Defeitos de membros: deformidades congênitas do pé (Q66), polidactilia (Q69), defeitos, por redução, do membro superior (Q71), defeitos, por redução, do membro inferior (Q72), defeitos por redução de membro não especificada (Q73) e artrogripose congênita múltipla (Q74.3).
7) Defeitos de parede abdominal: exonfalia (Q79.2) e gastrosquise (Q79.3).
8) Síndrome de Down (Q90).

As anomalias não classificadas pela lista foram agrupadas e denominadas “Não classificadas/demais”. 

### Fontes de dados e mensuração

A fonte de dados é o Sinasc da Secretaria Estadual de Saúde do Rio de Janeiro. As bases de dados secundários foram cedidas para a pesquisa sem identificação. A prevalência total de anomalias é o quociente entre o número de recém-nascidos com anomalias (campo 6 da Declaração de Nascido Vivo - DNV), assinalado “sim”, independentemente de ter o código da CID-10 no campo 41 da DNV) (17), e o total de nascidos vivos, multiplicado por 10 mil. A prevalência de anomalias congênitas prioritárias considerou o código da CID-10, podendo um mesmo recém-nascido ter anomalias classificadas em mais de um grupo. 

### Tamanho do estudo

De 2019 a 2021, foram registrados 598.604 nascidos vivos, sendo 597.535 (99,8%) elegíveis.

### Métodos estatísticos

As frequências de recém-nascidos elegíveis e não elegíveis segundo o preenchimento do campo 6 da DNV foram comparadas. Utilizou-se o teste exato de Fisher.

A frequência de anomalias por recém-nascido (até 5), as prevalências de anomalias totais e por variáveis selecionadas e os intervalos de confiança (IC95%) foram calculados.

## Resultados

Entre os 597.535 recém-nascidos elegíveis, 99,3% do campo 6 da DNV foram preenchidos, 2,6% ficaram na categoria “ignorado”, somando 3,3% de ausência da informação. Entre os não elegíveis (n=1.069), os valores correspondentes foram 99,2%, 3,9% e 4,7%. As diferenças de presença de anomalias segundo elegibilidade foram significantes (<0,005). As prevalências de anomalias por 10 mil nascidos vivos foram 68,7 (elegíveis) e 13,3 (não elegíveis) (Tabela 2). 

**Tabela 2:** Distribuição dos nascidos vivos segundo a presença e a frequência de anomalias congênitas (campos 6 e 41 da Declaração de Nascido Vivo). Rio de Janeiro, 2019-2021

Campos	2019	2020	2021	Total
	n (%)	n (%)	n (%)	n (%)
**Presença de anomalias congênitas**				
Sim	1.415 (0,7)	1.405 (0,7)	1.288 (0,7)	4.108 (0,7)
Não	202.196 (97,1)	190.528 (95,6)	181.045 (95,3)	573.769 (96,0)
Ignorado	4.379 (2,1)	6.274 (3,2)	4.739 (2,5)	15.392 (2,6)
Em branco	314 (0,2)	1.011 (0,5)	2.941 (1,6)	4.266 (0,7)
Total	208.304 (100,0)	199.218 (100,0)	190.013 (100,0)	597.535 (100,0)
**Frequência de anomalias congênitas**				
1 anomalia	1.161 (80,0)	1.157 (80,8)	1.047 (79,0)	3.365 (79,6)
2 anomalias	168 (11,6)	159 (11,1)	159 (12,0)	507 (12,0)
3 anomalias	83 (5,7)	68 (4,7)	81 (6,1)	232 (5,5)
4 anomalias	22 (1,5)	34 (2,4)	22 (1,7)	78 (1,8)
5 anomalias	17 (1,2)	14 (1,0)	17 (1,3)	48 (1,1)
Total	1.451 (100,0)	1.432 (100,0)	1.326 (100,0)	4.230 (100,0)

A seguir, os resultados correspondem aos recém-nascidos elegíveis. 

A prevalência de anomalia variou de 68 a 71/10 mil nascidos vivos. A frequência de apenas uma anomalia é predominante, variando de 79%, em 2021, a 80,8% em 2020. A frequência de anomalias vai gradativamente diminuindo, alcançando 1,1% para a categoria de cinco anomalias (Tabela 2). 

Recém-nascidos de mães adolescentes (<20 anos) e, principalmente, aquelas com idade mais avançada, apresentaram prevalências mais elevadas de anomalias. Maiores prevalências também foram observadas entre os recém-nascidos de mulheres pretas e com baixa escolaridade, exceto em 2019, de gravidez múltipla, que realizaram cesariana e com pré-natal inadequado. A diferença entre as prevalências em primíparas e multíparas foi de 0,2/10 mil nascidos vivos. Recém-nascidos do sexo masculino e em situação de risco biológico (baixo peso, prematuridade e/ou asfixia) também apresentaram maiores prevalências. A magnitude das prevalências anuais de anomalias variou pouco, havendo sobreposição dos respectivos IC95% (Tabela 3).

**Tabela 3:** Frequência absoluta (n) e prevalência de anomalias congênitas por 10 mil nascidos vivos e respectivos intervalos de confiança de 95% (IC95%) total e anual, segundo características maternas, da gestação, do parto, da história reprodutiva, assistenciais e do recém-nascido. Rio de Janeiro, 2019-2021

Características	2019		2020		2021		Total	
	n	Prevalência (IC95%)	n	Prevalência (IC95%)	n	Prevalência (IC95%)	n	Prevalência (IC95%)
**Idade** (anos)								
<20	208	72,2 (63,0; 82,6)	209	78,7 (68,8; 90,1)	172	73,5 (63,4; 85,3)	589	74,8 (69,0; 81,1)
20-34	914	63,9 (59,9; 68,1)	884	64,2 (60,1; 68,6)	840	63,3 (59,1; 67,7)	2.638	63,8 (61,4; 66,3)
≥35	293	80,6 (72,0; 90,4)	312	89,1 (79,8; 99,6)	276	81,6 (72,5; 91,7)	881	83,8 (78,4; 89,5)
**Raça/cor da pele**								
Branca	493	70,7 (64,7; 77,2)	422	66,2 (60,2; 72,8)	370	61,4 (55,5; 68,0)	1.285	66,3 (62,8; 70.0)
Preta	189	68,9 (59,9; 79,5)	226	80,7 (70,9; 91,9)	221	81,0 (71,1; 92,4)	636	76,9 (71,2; 83,1)
Parda	674	63,0 (58,4; 67,9)	699	68,5 (63,6; 73,8)	648	66,9 (62,0; 72,2)	2.021	66,1 (63,3; 69,0)
**Anos de estudo**								
<4	16	57,1 (35,2; 92,5)	19	76,6 (49,1; 119,3)	21	99,2 (65,0; 151,2)	56	75,7 (58,3; 98,1)
4-11	1.063	67,3 (63,4; 71,5)	1.069	70,6 (66,5; 75,0)	1.002	70,3 (66,1; 74,8)	3.134	69,4 (67,0; 71,9)
≥12	294	67,2 (59,9; 75,3)	291	70,2 (62,6; 78,7)	241	58,5 (51,6; 66,3)	826	65,3 (61,0; 69,9)
**Gravidez**								
Única	1.371	67,4 (63,9; 71,0)	1.359	69,8 (66,2; 73,6)	1.262	68,0 (64,4; 71,9)	3.992	68,4 (66,3; 70,6)
Múltipla	41	86,2 (63,6; 116,7)	46	102,2 (76,7; 136,0)	24	54,9 (36,9; 81,5)	111	81,4 (67,7; 98,0)
**Paridade**								
Primípara	645	71,9 (66,5; 77,6)	547	65,9 (60,6; 71,6)	540	67,1 (61,7; 73,0)	1.732	68,4 (65,3; 71,7)
Multípara	761	65,3 (60,8; 70,0)	802	71,3 (66,6; 76,4)	734	68,0 (63,3; 73,1)	2.297	68,2 (65,4; 71,0)
**Tipo de parto**								
Vaginal	466	53,1 (48,5; 58,1)	463	56,1 (51,2; 61,4)	423	53,4 (48,6; 58,7)	1.352	53,4 (50,6; 56,3)
Cesariana	945	78,5 (73,7; 83,7)	942	80,8 (75,9; 86,1)	865	78,1 (73,1; 83,5)	2.752	81,7 (78,7; 84,8)
**Pré-natal**								
Adequado	918	63,4 (59,5; 67,7)	859	62,9 (58,8; 67,2)	810	61,2 (57,2; 65,6)	2.587	62,5 (60,2; 65,0)
Inadequado	377	76,7 (69,4; 84,8)	426	88,1 (80,2; 96,9)	380	85,7 (77,5; 94,7)	1.183	83,4 (78,8; 88,3)
**Sexo**								
Feminino	588	57,6 (53,2; 62,5)	587	60,4 (55,8; 65,5)	538	57,8 (53,1; 62,9)	1.713	58,6 (55,9; 61,5)
Masculino	809	76,2 (71,1; 81,6)	796	78,0 (72,8; 83,6)	728	75,1 (68,9; 80,8)	2.333	76,5 (73,4; 79,6)
**Peso** (gramas)								
<1.500	72	238,9 (190,1; 299,8)	87	291,9 (237,3; 358,7)	85	302,5 (245,3; 372,5)	244	277,1 (244,9; 313,5)
1.500-2.499	296	185,0 (165,3; 207,1)	272	177,0 (157,3; 199,0)	283	187,7 (167,2; 210,6)	851	183,2 (171,4; 195,8)
2.500-3.999	984	55,1 (51,8; 58,6)	985	58,0 (54,5; 61,7)	868	53,4 (50,0; 57,0)	2.837	55,5 (53,5; 57,6)
≥4.000	63	59,1 (46,2; 75,6)	61	55,8 (43,4; 71,6)	52	54,6 (41,7; 71,5)	176	56,6 (48,8; 65,5)
**Gestação** (semanas)								
22-31	61	189,1 (147,5; 242,2)	58	181,2 (140,4; 233,5)	59	198,0 (153,8; 254,5)	178	189,2 (163,6: 218,8)
32-36	271	140,9 (125,1; 158,5)	316	168,2 (150,8; 187,6)	285	155,7 (138,7; 174,6)	872	154,8 (144,9: 165,3)
≥37	1.061	57,8 (54,4; 61,4)	1.011	58,0 (54,5; 61,7)	922	55,7 (52,2; 59,4)	2.994	57,2 (55,2; 59,3)
**Apgar 5** ^o^ **minuto**								
<7	151	717,3 (614,7; 835,5)	140	672,8 (572,9; 788,5)	144	712,9 (608,6; 833,4)	435	700,9 (640,0; 767,1)
≥7	1.246	61,1 (57,8; 64,6)	1.249	64,0 (60,6; 67,7)	1.136	61,0 (57,6; 64,7)	3.631	62,1 (60,1; 64,1)

No triênio, entre os 4.108 recém-nascidos com anomalia descrita, 2.739 (66,7%) foram classificadas pela lista de anomalias prioritárias, correspondendo a uma prevalência de 45,8/10 mil nascidos vivos, duas vezes superior à prevalência das anomalias não classificadas (22,9/10 mil nascidos vivos). O padrão da prevalência de anomalias congênitas prioritárias se mantém anualmente. Valores mais elevados foram observados para os defeitos de membros, de 3,8 a 4,6 vezes o valor anual da segunda posição, as cardiopatias congênitas, em todos os anos analisados, exceto em 2021, em que fendas orais ultrapassam as cardiopatias. Nos demais anos, a terceira, quarta e quinta posições se alternam em fendas orais, anomalias de órgãos genitais e defeitos de parede abdominal (Tabela 4). 

**Tabela 4:** Frequência absoluta (n) de anomalias congênitas e respectivas prevalências por 10 mil nascidos vivos, com os intervalos de confiança de 95% (IC95%), segundo a lista de anomalias congênitas prioritárias para vigilância no âmbito do Sistema de Informação sobre Nascidos Vivos. Rio de Janeiro, 2019-2021

Anomalias congênitas	2019		2020		2021		Total	
	n	Prevalência (IC95%)	n	Prevalência (IC95%)	n	Prevalência (IC95%)	n	Prevalência (IC95%)
**Anomalias prioritárias**								
1) Defeitos do tubo neural	71	3,4 (2,7; 4,3)	71	3,6 (2,8; 4,5)	72	3,8 (3,0; 4,8)	214	3,6 (3,1; 4,1)
2) Microcefalia	12	0,6 (0,3; 1,0)	19	1,0 (0,6; 1,5)	18	0,9 (0,6; 1,5)	49	0,8 (0,6; 1,1)
3) Cardiopatias congênitas	116	5,6 (4,6; 6,7)	120	6,0 (5,0; 7,2)	92	4,8 (3,9; 5,9)	328	6,5 (5,9; 7,2)
4) Fendas orais	86	4,1 (3,3; 5,1)	89	4,5 (3,6; 5,5)	95	5,0 (4,1; 6,1)	270	4,5 (4,0; 5,1)
5) Anomalias de órgãos genitais	94	4,5 (3,7; 5,5)	86	4,3 (3,5; 5,3)	72	3,8 (3,0; 4,8)	252	4,2 (3,7; 4,8)
6) Defeitos de membros	446	21,4 (19,5; 23,5)	463	23,2 (21,2; 25,5)	434	22,8 (20,8; 25,1)	1.343	22,5 (21,3; 23,7)
7) Defeitos de parede abdominal	86	4,1 (3,3; 5,1)	88	4,4 (3,6; 5,4)	70	3,7 (2,9; 4,7)	244	4,1 (3,6; 4,6)
8) Síndrome de Down	56	2,7 (2,1; 3,5)	62	3,1 (2,4; 4,0)	66	3,5 (2,7; 4,4)	184	3,1 (2,7; 3,6)
**Pelo menos uma anomalia prioritária**	924	44,4 (41,6; 47,3)	949	47,6 (44,7; 50,8)	866	45,6 (42,7; 48,7)	2.739	45,8 (44,2; 47,6)
**Anomalia não classificada**	491	23,6 (21,6; 25,8)	456	22,9 (20,9; 25,1)	422	22,2 (20,2; 24,4)	1.369	22,9 (21,7; 24,2)

Considerando-se que ocorreram pequenas variações anuais de anomalias, os resultados a seguir são apresentados apenas para o triênio (Tabela 5). 

**Tabela 5:** Distribuição absoluta (n) e percentual (%) dos nascidos vivos com anomalias congênitas e sem anomalia, segundo a lista de anomalias congênitas prioritárias para vigilância no âmbito do Sistema de Informação sobre Nascidos Vivos, por características maternas, da gestação, do parto, da história reprodutiva, assistenciais e do recém-nascido. Rio de Janeiro, 2019-2021

Características	Defeito do tubo neural n (%)	Microcefalia n (%)	Cardiopatias n (%)	Fendas orais n (%)	Órgãos genitais n (%)	Membros n (%)	Parede abdominal n (%)	Síndrome de Down n (%)	Sem anomalia n (%)
**Idade** (anos)									
<20	24 (11,2)	8 (16,3)	26 (7,9)	24 (8,9)	39 (15,5)	195 (14,5)	88 (36,1)	10 (5,4)	76.129 (13,3)
20-34	144 (67,3)	32 (65,3)	204 (62,2)	179 (66,3)	159 (63,1)	925 (68,9)	133 (54,5)	63 (34,2)	398.127 (69,4)
≥35	46 (21,5)	9 (18,4)	98 (29,9)	67 (24,8)	54 (21,4)	223 (16,6)	23 (9,4)	111 (60,3)	99.491 (17,3)
**Raça/cor da pele**									
Branca	64 (33,2)	21 (45,7)	143 (46,3)	92 (35,0)	79 (32,0)	352 (26,8)	60 (27,8)	85 (48,0)	184.901 (32,9)
Preta	30 (15,5)	7 (15,2)	40 (12,9)	34 (12,9)	45 (18,2)	251 (19,1)	31 (14,4)	16 (9,0)	80.396 (14,3)
Parda	99 (51,3)	18 (39,1)	126 (40,8)	137 (52,1)	123 (49,8)	711 (54,1)	125 (57,9)	76 (42,9)	296.344 (52,8)
**Anos de estudo**									
<4	5 (2,4)	0	1 (0,3)	3 (1,1)	3 (1,2)	18 (1,4)	1 (0,4)	2 (1,1)	7.216 (1,3)
4-11	158 (76,0)	44 (89,8)	195 (60,4)	201 (75,6)	203 (82,5)	1074 (81,8)	216 (88,9)	111 (62,0)	437.095 (77,6)
≥12	45 (21,6)	5 (10,2)	127 (39,3)	62 (23,3)	40 (16,3)	221 (16,8)	26 (10,7)	66 (36,9)	118.747 (21,1)
**Gravidez**									
Única	205 (95,8)	49 (100,0)	316 (96,3)	268 (99,3)	245 (97,2)	1314 (97,9)	235 (96,3)	181 (98,4)	560.452 (97,7)
Múltipla	9 (4,2)	0	12 (3,7)	2 (0,7)	7 (2,8)	28 (2,1)	9 (3,7)	3 (1,6)	13.069 (2,3)
**Paridade**									
Primípara	82 (39,4)	19 (40,4)	155 (48,1)	98 (36,6)	132 (52,6)	569 (42,7)	137 (59,8)	67 (36,6)	243.823 (42,9)
Multípara	126 (60,6)	28 (59,6)	167 (51,9)	170 (63,4)	119 (47,4)	765 (57,3)	92 (40,2)	116 (63,4)	324.229 (57,1)
**Tipo de parto**									
Vaginal	48 (22,4)	11 (22,4)	54 (16,5)	86 (33,5)	90 (33,5)	547 (33,5)	55 (33,5)	47 (33,5)	241.946 (33,5)
Cesariana	166 (77,6)	38 (77,6)	274 (83,5)	184 (63,4)	162 (63,4)	793 (63,4)	189 (63,4)	137 (63,4)	331.572 (63,4)
**Pré-natal**									
Adequado	117 (61,3)	28 (60,9)	250 (80,6)	182 (73,1)	160 (69,9)	825 (67,0)	127 (56,4)	143 (81,3)	399.007 (74,4)
Inadequado	74 (38,7)	18 (39,1)	60 (19,4)	67 (26,9)	69 (30,1)	406 (33,0)	98 (43,6)	33 (18,8)	137.258 (25,6)
**Sexo**									
Feminino	111 (52,9)	29 (60,4)	166 (50,9)	11 (6,6)	4 (2,1)	531 (39,9)	101 (41,9)	92 (50,0)	281.052 (49,0)
Masculino	99 (47,1)	19 (39,6)	160 (49,1)	156 (93,4)	186 (97,9)	799 (60,1)	140 (58,1)	92 (50,0)	292.717 (51,0)
**Peso** (gramas)									
<1.500	33 (15,4)	3 (6,1)	28 (8,5)	14 (5,2)	27 (10,7)	43 (3,2)	23 (9,4)	3 (1,6)	8.307 (1,4)
1.500-2.499	56 (26,2)	19 (38,8)	78 (23,8)	51 (18,9)	70 (27,8)	147 (10,9)	129 (52,9)	37 (20,1)	44.174 (7,7)
≥2.500	125 (58,4)	27 (55,1)	222 (67,7)	205 (75,9)	155 (61,5)	1.153 (85,9)	92 (37,7)	144 (78,3)	521.288 (90,9)
**Gestação** (semanas)									
22-31	22 (10,5)	1 (2,1)	13 (4,0)	9 (3,4)	19 (7,7)	36 (2,7)	20 (8,4)	3 (1,6)	8.876 (1,6)
32-36	45 (21,4)	14 (29,2)	87 (27,1)	44 (16,5)	61 (24,6)	178 (13,4)	105 (44,1)	41 (22,4)	53.595 (9,5)
≥37	143 (68,1)	33 (68,8)	221 (68,8)	214 (80,1)	168 (67,7)	1.115 (83,9)	113 (47,5)	139 (76,0)	504.495 (89,0)
**Apgar 5** ^o^ **minuto**									
<7	59 (28,0)	15 (31,3)	43 (13,1)	23 (8,6)	33 (13,3)	67 (5,0)	32 (13,2)	5 (2,7)	5.569 (1,0)
≥7	152 (72,0)	33 (68,8)	284 (86,9)	244 (91,4)	215 (86,7)	1.263 (95,0)	210 (86,8)	178 (97,3)	562.317 (99,0)

Entre os recém-nascidos com anomalias prioritárias de notificação e aqueles sem anomalias, predominaram mães entre 20 e 34 anos, seguidos daquelas com 35 anos e mais. As exceções ocorreram para aqueles que apresentaram deficiência de parede abdominal, em que mães adolescentes (36,1%) prevalecem; e para os que apresentaram síndrome de Down, em que predominam mães com idade ≥35 anos (60,3%). Houve uma maior frequência de mães de cor parda nos resultados gerais, exceto nos casos de recém-nascidos com microcefalia ou cardiopatias, em que a frequência de mães de cor branca foi superior à de mães pardas (Tabela 4). 

A faixa de 4 a 11 anos de estudo predominou entre as mães dos recém-nascidos, independentemente da presença de anomalia, seguida da faixa ≥12 anos. Observa-se que mães de recém-nascidos com cardiopatias e com síndrome de Down têm percentuais mais elevados de maior escolaridade (39,3% e 36,9%, respectivamente), enquanto mães de recém-nascidos com microcefalia e defeitos de parede abdominal têm percentuais bem inferiores nesta categoria (10,2% e 10,7%, respectivamente), e mães de recém-nascidos com defeitos do tubo neural apresentam o maior percentual de baixa escolaridade (2,4%) (Tabela 5).

A gravidez única foi a mais frequente, independentemente da presença ou do tipo de anomalia classificada pela lista de anomalias prioritárias. Nenhuma mãe de recém-nascido com microcefalia teve gravidez múltipla. Primíparas predominaram apenas entre recém-nascidos com anomalias de órgãos genitais e defeitos de parede abdominal (Tabela 5).

A cesariana foi mais frequente, independentemente da presença de anomalias, com percentuais mais elevados entre as mulheres cujos neonatos apresentaram cardiopatias, defeitos do tubo neural e microcefalia. As maiores frequências de inadequação de pré-natal ocorreram entre as mães de recém-nascidos com defeitos de parede abdominal, defeitos de tubo neural e microcefalia (Tabela 5).

A maioria das anomalias foi mais frequente em meninos, exceto defeitos de tubo neural, microcefalia e cardiopatias, mais comum em meninas, e síndrome de Down, sem diferença. Destacaram-se as anomalias genitais, quase na totalidade entre meninos. A frequência de baixo peso foi elevada em todos os grupos de anomalias prioritárias, e aqueles com defeitos do tubo neural, anomalias de órgãos genitais e defeitos de parede abdominal alcançaram maior frequência de peso <1.500g. Idade gestacional <37 semanas também foi de maior frequência entre aqueles com anomalia. A maior frequência de prematuridade foi registrada em recém-nascidos com defeitos de parede abdominal, anomalias genitais, defeitos do tubo neural e cardiopatias. A asfixia apresentou uma elevada proporção entre recém-nascidos com microcefalia e defeitos do tubo neural (25,1%). Entre os recém-nascidos sem anomalia, este percentual é de 1% (Tabela 5). 

Dos 4.108 recém-nascidos com anomalia, 34,1% (1.369) não foram classificados segundo a lista de anomalias prioritárias. A maioria das anomalias não classificadas é única (n=1.119; 81,7%), sendo as mais frequentes: malformações congênitas múltiplas não diagnosticadas em outra parte (Q89.7; n=83), hidrocefalia congênita do encéfalo não especificada (Q03.9; n=58), hérnia diafragmática congênita (Q79.0; n=40), malformação congênita não especificada (Q89.9; n=37), e malformação congênita não especificada da orelha (Q17.9; n=36). Seis recém-nascidos apresentaram anomalia não classificada do capítulo II-Neoplasias da CID-10 (D18.0 - hemangioma).

## Discussão

No Rio de Janeiro, de 2019 a 2021, a prevalência de anomalias foi maior em recém-nascidos com maior risco biológico (baixo peso, prematuridade e asfixia), filhos de mulheres com maior vulnerabilidade sociodemográfica (extremos etários, pretas, baixa escolaridade) e com realização inadequada de pré-natal. Considerando-se o nível de desenvolvimento do estado, identificaram-se desigualdades sociais e de acesso a serviços de saúde na prevalência de anomalias. 

As múltiplas relações entre as variáveis e os potenciais confundidores, não contemplados, além da natureza transversal deste estudo, limitam a interpretação causal dos resultados. Quanto à validade do diagnóstico da anomalia congênita, sabe-se que, quanto maior a complexidade da anomalia, maior o risco de subnotificação e de incompletude dos dados (15). A lista de anomalias prioritárias contempla a maioria das anomalias congênitas, porém, ainda não foi incorporada às tabulações disponibilizadas pelo Departamento de Informática do Sistema Único de Saúde, fator limitante para sua utilização.

No Brasil e na região Sudeste, de 2001 a 2018, a tendência da prevalência de anomalia mostrou um crescimento anual de respectivamente 5,2% e 5,7%, sendo atribuído esse aumento ao modelo vigente da DNV desde 2011, por permitir maior captação da informação sobre anomalia, e à epidemia de Zika vírus de 2015 a 2016 (16). A prevalência de anomalia por 10 mil nascidos vivos foi 87,2 no país, de 2018 a 2020 (3), e de 93,0 no Rio Grande do Sul, de 2012 a 2015 (7), valores superiores ao do Rio de Janeiro no presente estudo. Por sua vez, em Tangará da Serra, Mato Grosso, a prevalência foi inferior, cerca de 49/10 mil nascidos vivos (17). Quanto à prevalência de anomalias prioritárias, os valores do Rio Grande do Sul de 2010 a 2019 também foram superiores ao do Rio de Janeiro de 2019 a 2021, 60,7/10 mil nascidos vivos (18).

Entre os recém-nascidos com anomalias analisados neste estudo, 79,6% apresentaram uma única anomalia, valor próximo ao do Brasil de 2018 a 2020 (81,3%), (3). 

Quanto ao perfil dos recém-nascidos com anomalia, os resultados encontrados convergiram maioritariamente com os de outros estudos nacionais que utilizaram o Sinasc. As prevalências de maior magnitude no país correspondem a recém-nascidos filhos de mulheres pretas, com idade >30 anos, consultas de pré-natal <7, meninos e com peso <1.500g, prematuros, com asfixia (3), resultados semelhantes aos encontrados para o estado do Rio de Janeiro. Em Santa Catariana, de 2010 a 2018, e no município de São Paulo, de 2010 a 2014, a ocorrência de anomalias também esteve associada com baixo peso, prematuridade, baixo escore de Apgar (19-20) e nenhuma consulta de pré-natal (7). Quanto à escolaridade materna, encontramos maior prevalência na faixa <4 anos de estudo, em concordância com a razão de chances de anomalia estimada no Rio Grande do Sul (<4 comparadas com ≥12 anos) (7). Diferentemente, no país e em Tangará da Serra, maiores prevalências de anomalia ocorreram entre mães com escolaridade ≥12 anos (3,17). Foram encontrados poucos estudos utilizando a lista de anomalias prioritárias (18). 

As anomalias de membros se caracterizam pela ausência ou hipoplasia grave de um membro completo ou parte dele, sendo comum a coexistência de outras anomalias (18). No estado do Rio de Janeiro, destacaram-se os defeitos de membros como o principal grupo da lista, correspondendo à prevalência de 22,5/10 mil nascidos vivos, valor 3,5 vezes o da prevalência das cardiopatias, que ocupam a segunda posição. Vale ressaltar que os defeitos de membros são de grande facilidade diagnóstica ao nascimento, o que nem sempre ocorre com as cardiopatias (2). Entre os recém-nascidos com defeitos de membros, predominaram aqueles com menor risco biológico (peso ≥2.500g, a termo e sem asfixia). 

As cardiopatias são as anomalias mais comuns, embora, quando aferidas ao nascimento, possam estar subestimadas (18). Doenças maternas preexistentes, como diabetes e colagenoses, bem como as adquiridas durante a gestação, a exemplo das infecções virais e parasitárias, medicamentos, fatores comportamentais (uso de drogas lícitas e ilícitas) e idade materna avançada representam, entre outros, fatores de risco materno para cardiopatias (18). Entre esses fatores mencionados, apenas a informação sobre idade materna consta no Sinasc. No estado do Rio de Janeiro, entre os recém-nascidos com cardiopatias notificadas ao nascimento, 30% das mães tinham idade ≥35 anos, e no município de São Paulo, a maior prevalência de cardiopatias foi registrada nessa faixa etária (21). A prevalência de cardiopatias por 10 mil nascidos vivos no país variou entre 5,1 e 10,^8^, e no estado do Rio de Janeiro, entre 2,9 e 5,^7^, de 2010 a 2019 (18). No estado de São Paulo, alcançou 12,4/10 mil nascidos vivos, com tendência ascendente (21). Nossos resultados mostraram a prevalência de cardiopatias de 6,5/10 mil nascidos vivos, e o perfil dos recém-nascidos com cardiopatias foi semelhante ao do país (18).

Seguindo a ordem de frequência das anomalias prioritárias, estão as fendas orais, anomalias de órgãos genitais e os defeitos de parede abdominal, com prevalências próximas entre si, respectivamente, 4,^5^, 4,2 e 4,1/10 mil nascidos vivos.

As fendas orais são comuns entre as anomalias e em geral são diagnosticadas pelo exame físico ao nascimento, embora possam ser detectadas por ultrassonografia no pré-natal (18). No estado do Rio de Janeiro, observou-se um aumento gradativo da prevalência anual de fendas orais, principalmente em 2020 e 2021 (pandemia de covid-19), quando comparadas a 2019, embora com sobreposição dos IC95%. Observou-se um comportamento semelhante com as prevalências de anomalias do tubo neural, quinta posição. Problemas com a assistência pré-natal durante a pandemia podem ter afetado o cumprimento de medidas preventivas (22). A investigação das consequências fetais da infecção do Sars-Cov-19 na gestação ainda está em andamento. Particularmente em relação às fendas orais, um estudo retrospectivo e controlado de base hospitalar encontrou associação entre fenda palatina não sindrômica do recém-nascido e covid-19 na gestação, porém não foi uma análise ajustada para confundimento (20). 

O grupo de anomalias de órgãos genitais corresponde às desordens de diferenciação sexual (18). No Brasil, a prevalência foi 4,7/10 mil nascidos vivos (de 2010 a 2019) e o perfil materno foi semelhante ao do estado do Rio de Janeiro de 2019 a 2021: mães jovens, pardas e com escolaridade média. No Distrito Federal, de 2010 a 2021, esse grupo alcançou a segunda posição (10,3/10 mil nascidos vivos), perdendo apenas para defeito de membros (21,8/10 mil nascidos vivos) (23). O sub-registro de anomalias de órgãos sexuais é relatado em algumas publicações nacionais quando comparado a valores europeus (18,24).

Os defeitos de parede abdominal são caracterizados pela herniação dos órgãos abdominais (18). Podem ser diagnosticadas no pré-natal e são visíveis ao nascimento (25). Um fator de risco conhecido para essas anomalias é a idade materna jovem (9,26). No presente estudo, observou-se que recém-nascidos de mães adolescentes têm maior prevalência de defeitos da parede abdominal quando comparados aos demais grupos de anomalias prioritárias e aqueles sem anomalias.

O grupo de anomalias prioritárias com menor prevalência neste estudo foi a microcefalia (0,8/10 mil nascidos vivos). No Sinasc, somente as microcefalias graves são notificadas (perímetro cefálico <3 desvios-padrão) (27). No Brasil, e em particular no estado do Rio de Janeiro, as prevalências anuais de microcefalia por 100 mil nascidos vivos, de 2010 a 2019, foram inferiores a 1, exceto em 2015 e 2016 (epidemia de Zika vírus), quando os valores alcançaram, respectivamente, cerca de 6 e 8 no país e 2,1 e 10,6 no estado (18). No país, as características das mães e dos recém-nascidos com microcefalia foram consistentes com as observadas neste estudo de 2019 a 2021, com exceção da maior prevalência de mães pardas no contexto nacional em comparação com mães brancas no estado do Rio de Janeiro, e uma maior incidência de asfixia, que foi de 12,2% no Brasil e de aproximadamente 31% no Rio de Janeiro (18). 

A incorporação de microcefalia na lista de anomalias prioritárias deveu-se ao fato de ser uma anomalia sentinela para infecções congênitas, como Zika vírus e citomegalovírus (2). Em julho de 2024, o Ponto Focal Nacional do Regulamento Sanitário Internacional no Brasil informou à Organização Pan-Americana da Saúde (Opas) sobre a suspeita de transmissão vertical do vírus Oropouche (OROV) (28). No Brasil, foi realizada uma investigação retrospectiva de amostras de soro e líquido cefalorraquidiano com resultado negativo para dengue, chikungunya, Zika e vírus do Nilo Ocidental. Foram detectados anticorpos IgM para o OROV no soro e líquido cefalorraquidiano dos quatro recém-nascidos com microcefalia (28). Nesse sentido, a Opas solicitou aos Estados-membros com transmissão comprovada de OROV ou outros arbovírus que intensifiquem a vigilância em gestantes e notifiquem a ocorrência de aborto espontâneo/óbito fetal associados à infecção por OROV, bem como o aumento desses e de malformações congênitas em recém-nascidos que não possam ser explicadas por uma causa conhecida (28).

A síndrome de Down é a síndrome genética mais comum e apresenta sinais e sintomas que, em geral, são reconhecidos ao nascimento, sendo algumas passíveis de diagnóstico no pré-natal (2,18). A idade materna avançada aumenta o risco de anomalias cromossômicas, incluindo a síndrome de Down (1), sendo comum a concomitância com cardiopatias congênitas (18). No presente estudo, a maior frequência de mães ≥35 anos foi observada apenas para aqueles com síndrome de Down (60,3%), quando comparada aos demais grupos e sem anomalia. 

Medidas de prevenção de anomalias congênitas incluem o cuidado pré-concepcional de mulheres com condições de saúde crônicas que possam afetar a embriogênese ou o desenvolvimento fetal, o cuidado pré-natal, e o cuidado multidisciplinar com a saúde das crianças com anomalias (1,29).

A importância da vigilância de anomalias congênitas com os dados do Sinasc e sua análise para a produção de informações qualificadas são primordiais para o direcionamento de ações de saúde, ainda mais se considerando o constante aprimoramento do Sinasc. Como ponto forte do estudo, destacamos a ótima completude dos campos 6 e 41 da DNV, em concordância com o encontrado para o país, apesar das diferenças regionais (30). Com o alerta da possível relação entre a infecção por OROV e anomalias, deve-se intensificar a vigilância das anomalias pelos sistemas de informações em saúde. A lista de anomalias prioritárias amplia o conhecimento das anomalias e auxilia nas ações de saúde, devendo ser implementada nos programas de tabulações de dados para uso rotineiro pelos servidores de saúde, pesquisadores e gestores. 

Conclui-se que o estudo identificou a prevalência e as características sociodemográficas das anomalias congênitas no estado do Rio de Janeiro, que não haviam sido descritas nos últimos anos. O padrão encontrado foi semelhante ao nacional quanto à ordem de frequência das anomalias congênitas e às desigualdades na sua ocorrência. O estudo servirá como linha de base para o monitoramento da prevalência de anomalias prioritárias no estado do Rio de Janeiro. 

## Data Availability

Os dados são de acesso público, e disponibilizados pela Secretaria Estadual de Saúde do Rio de Janeiro.
